# Role of *keratin 24* in human epidermal keratinocytes

**DOI:** 10.1371/journal.pone.0174626

**Published:** 2017-03-31

**Authors:** Min Min, Xi-Bei Chen, Ping Wang, Lilla Landeck, Jia-Qi Chen, Wei Li, Sui-Qing Cai, Min Zheng, Xiao-Yong Man

**Affiliations:** 1 Department of Dermatology, Second Affiliated Hospital, Zhejiang University School of Medicine, Hangzhou, China; 2 Ernst von Bergmann General Hospital, Teaching Hospital of Charité, Humboldt University, Potsdam, Germany; San Gallicano Dermatologic Institute, ITALY

## Abstract

*Keratin 24 (K24)* is a new kind of keratin genes, which encodes a novel keratin protein, K24 that bears high similarity to the type I keratins and displays a unique expression profile. However, the role of K24 is incompletely understood. In our study, we investigated the localization of K24 within the epidermis and possible functions. Keratin 24 was found to be modestly overexpressed in senescent keratinocytes and was mainly restricted to the upper stratum spinosum of epidermis. The protein was required for terminal differentiation upon CaCl_2_-induced differentiation. In vitro results showed that increased K24 in keratinocytes dramatically changed the differentiation of primary keratinocytes. It also inhibited cell survival by G1/S phase cell cycle arrest and induced senescence, autophagy and apoptosis of keratinocytes. In addition, K24 activated PKCδ signal pathway involving in cellular survival. In summary, K24 may be suggested as a potential differentiation marker and anti-proliferative factor in the epidermis.

## Introduction

The epidermis is a stratified epithelial tissue mainly built by keratinocytes that forms the outer skin layer and provides a physical barrier for human body by protecting the organism from environmental insults[1]. The life-cycle in a human keratinocyte, starting from the initial cell division at the basal epidermal layer until the uppermost cornified layer, is commonly completed in about 30 days[2].Throughout this period of time, the keratinocytes undergo fundamental changes in gene expression pattern, morphology and metabolism. Once the cells reach a state of terminal differentiation, they will only keep structural elements as mechanical barrier[3]. At present, an increasing number of studies conducted on in vitro skin models and in transgenic mice revealed a close relationship between epidermis and keratin[4–6].

Keratin intermediate filament (IF) proteins are epithelial cell cytoskeletal components that provide mechanical stability and protection from cell stress[7]. Keratin proteins take part in the formation of the IFs in epithelial cells and exist as polymeric filaments by pairing of type I (K1-K8, K71-K86) and type II (K9-K28, K31-K40) keratin proteins[8]. They are able to quickly responding to their cellular environment and can be up-regulated and/or modulated when encountered from cell stress. In addition, keratins function in a multitude of biological processes ranging from transcription regulation, proliferation, angiogenesis, adhesion, migration, epithelial polarity and inflammatory regulation to protein catabolism in various cellular compartments from extracellular to the nucleus[7].

Keratin 24 (K24; gene name *KRT24* or *FLJ20261* in humans; *Krt24*in mice), a cytokeratin-like protein of 525 amino acids, belongs to type I keratin polymers[9]. K24is reported to be highly expressed in keratinocytes, placenta, colon, and spleen[9].In humans, it is also suggested that *K24*might potentially serve as a susceptibility gene for early onset colorectal cancer [9, 10].On the contrary, Nieto-Miguel et al. described KRT24 as a terminally differentiated gene in corneal epithelia[11]. However, the functional roles of K24 within the epidermis are still unknown. Our study aimed to define the role of K24 in the biology of epidermal keratinocytes.

## Results

### K24 plays an important role in the differentiation of NHEK

**Fig 1:**
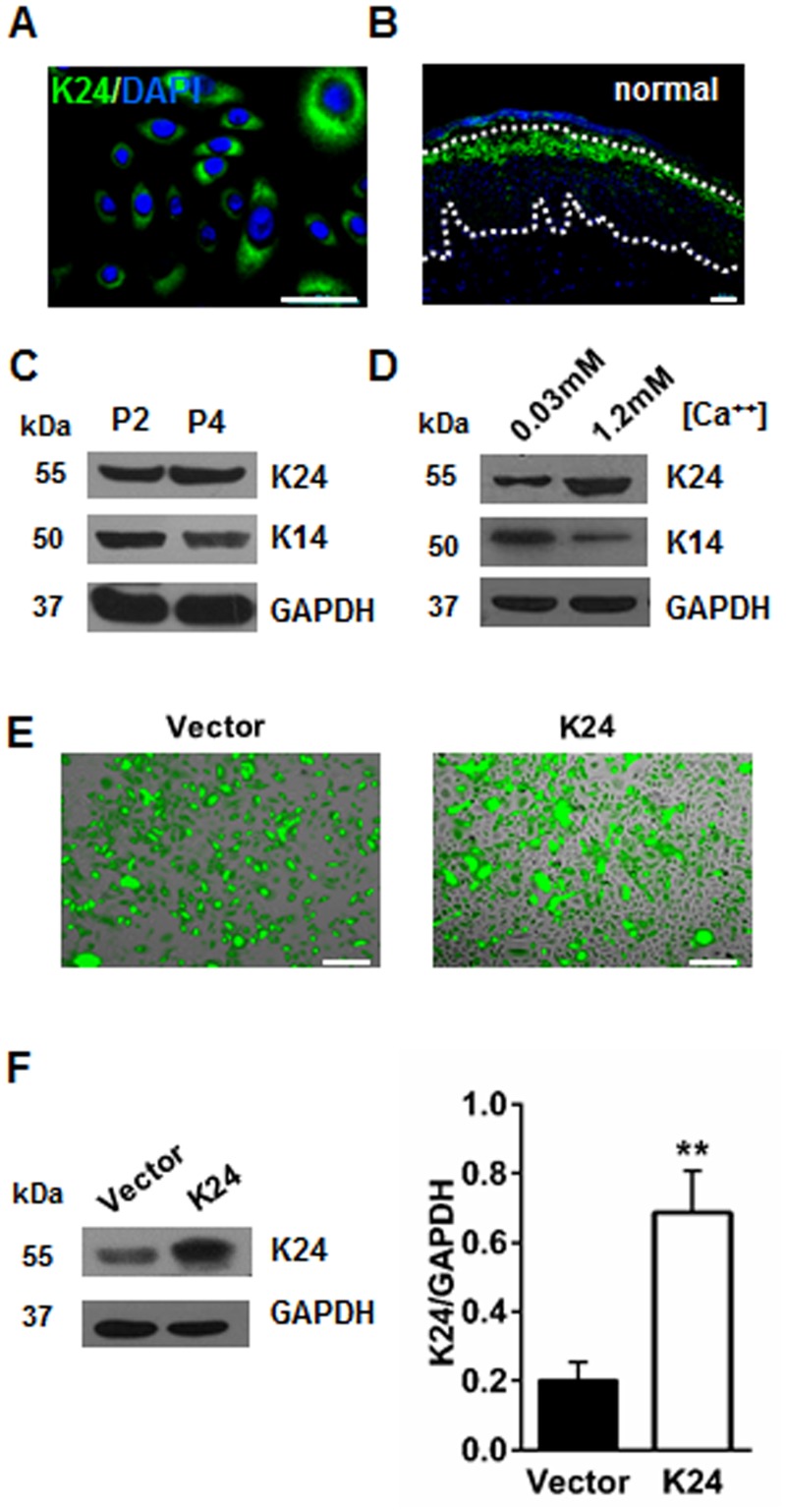
K24 plays an important role in the differentiation of NHEK. (A) Immunofluorescent staining of NHEK by K24 antibody. Green: K24; Blue: DAPI; Scale bar = 50 μm. (B) Immunofluorescent staining of normal epidermis by K24 antibody. Scale bar = 50 μm. Negative control images in which the primary antibody for rabbit IgG was used only are shown in S1 Fig. Enlarged immunofluorescent image of normal epidermis stained by K24 antibody is shown in S2 Fig. (C) Western blot analysis of K24 and K14 protein levels in subcultured 2^nd^ passage(P2) and 4^th^ passage(P4) primary keratinocytes. Similar results were obtained in three independent experiments. (D) Western blot analysis of K24 and K14 protein levels in NHEK following differentiation in 1.2 mM (high Ca+) and 0.03mM CaCl_2_(low Ca+) for 4 days. Similar results were observed in three independent experiments. (E)Pictures taken at 24h after K24 lentivirus transfection in NHEK cells. The transfection rate was over 80% in all experiments as demonstrated by GFP production. Scale bar = 50μm. (F)K24 protein levels in NHEK transfected with control vector and K24 overexpression vector were measured by western blot analysis. Quantification of K24 protein level in two groups are shown in the histogram. Vector: vector control group, K24: K24 overexpression group. GAPDH was used as the internal control. Data were expressed as mean ± s.e.m. Student t-test was used, **p<0.01vs vector control. The experiment was repeated with primary keratinocytes derived from five different donors.

Upon immunofluorescence, the expression of K24 was found mainly in the cytoplasm. In relative large keratinocytes, K24 became richer (Fig 1A). Regards the distribution of K24 within the epidermis, it was mainly localized in the upper stratum spinosum of normal epidermis, and the expression of K24 in basal layer and stratum spinosum is relative lower (Fig 1B). We next characterized the expression of K24 on subcultured keratinocyte and calcium-inducible differentiation. To evaluate the impact of K24 in subculture induced NHEK differentiation, the expression of basal epidermal marker K14 [12]and K24 in cell extracts prepared at passenger P2 and P4 were compared. Western blotting showed that the protein level of K24 was slightly increased in P4 cells, whereas K14 protein level was decreased (Fig 1C). K14 and K24 in whole cell protein extracts prepared at day 4 after the cells at passenger 2 being treated with calcium chloride at 0.03 mM and 1.2 mM separately, also showed increase of K24 and decrease of K14, a marker of mitotically active basal layer cells (Fig 1D).

### Overexpression of K24 induces inhibition of proliferation, cell cycle arrest and apoptosis in NHEK

**Fig 2:**
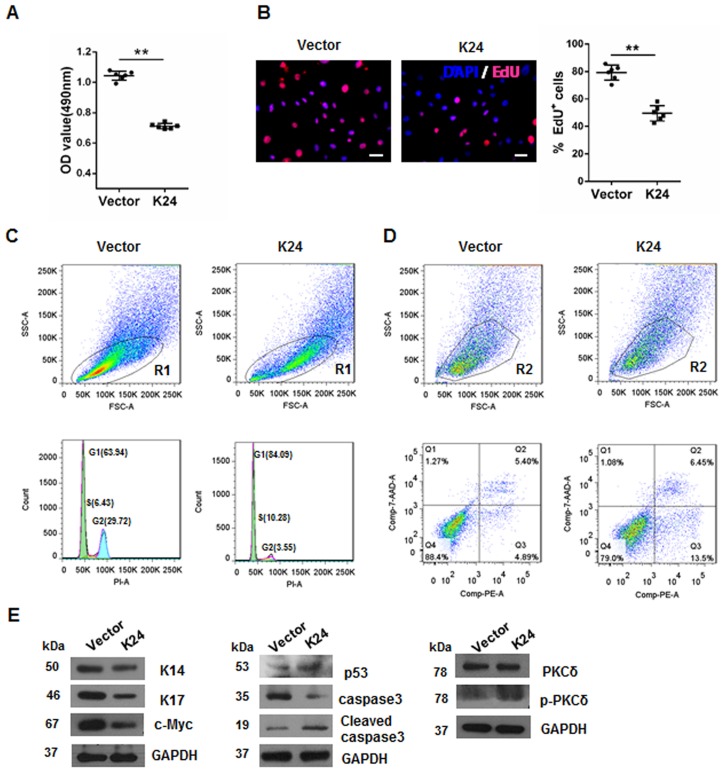
Overexpression of K24 in NHEK reduces cell growth, blocks cell cycle progression, and induces apoptosis. (A)Effect of K24 overexpression at 24h after transfection on cell proliferation measured by MTS assay (n = 6). Similar results were observed in three independent experiments. (B)Representative pictures of EdU incorporation in NHEK transfected with either vector or K24 lentivirus are shown. The values of EdU+% cells were determined in histogram. EdU is in red and Hoechst is in blue. Scale Bar = 100 μm. n = 6. The experiments were repeated three times with similar results. In (A) and (B), the Student t-test was used, **p<0.01 vs the vector control. (C)Representative pictures shows effect of K24 overexpression on cell cycle measured by cytometry analysis. Cells that fall into the R1 gate were selected for cell cycle analysis of total cells. Similar results were obtained in three independent experiments. (D)Representative pictures shows effect of K24 overexpression on the cell apoptosis rate determined by flow cytometry. Cell populations were gated (R2) as shown, the annexin V-positive cells are the cells undergoing apoptosis and are represented in the lower right quadrant. Similar results were obtained in three independent experiments. (E)Effect of K24 overexpression on the levels of K14, K17, c-Myc, p53, caspase3, PKCδ and p-PKCδ were measured by Western blot analysis with GAPDH protein as the internal control. The experiment was repeated with primary keratinocytes derived from three different donors.

Twenty-four hours after K24 lentivector (LV) and control LV transfection, the transfection rate in both groups was more than 80%, as determined by immunofluorescence (Fig 1E). The protein level of K24 in K24 LV-transfected keratinocytes increased more than three times as compared to that of scrambled vector-transduced keratinocytes (Fig 1F). To make clear the role of K24 in the metabolic activity of keratinocytes, MTS assay was performed. The results indicated that K24 overexpressed keratinocytes showed decreased proliferation rate, as compared with control cells (Fig 2A). Furthermore, the actual proliferative capacity of the K24 overexpressed keratinocytes was then determined by EdU assay. The percentage of proliferating cells from the K24 overexpressed cells was 43% after 72 h of incubation, whereas 78% being detected for the control cells (Fig 2B). In order to understand the underlying mechanism of K24-induced cell growth inhibition, cell cycle analysis was performed by FACS analysis. The result showed that the transfection of K24 decreased the DNA synthesis and induced a G1/S growth phase arrest, as evidenced by 84.09% in the experimental group, and 63.94% in the vector control cells (Fig 2C). We further analyses the apoptosis of above cells through FACS. Data showed that the population of annexin V single positive early apoptotic cells were markedly increased in K24 overexpression group (13.5% versus 4.89% of vector control cells) (Fig 2D). Furthermore, by using western blotting analysis, a decreased level of epidermal stem cell markers, K17, K14 and self-renewal marker c-Myc in K24 overexpressed keratinocytes were observed, as compared with control cells (Fig 2E). In addition, p53, Caspase-3 and p-PKCδ were profoundly up-regulated in K24 transfected cells (Fig 2E).

### K24 overexpression induces the overexpression of cytoskeletal protein, senescence and differentiation in NHEK

**Fig 3:**
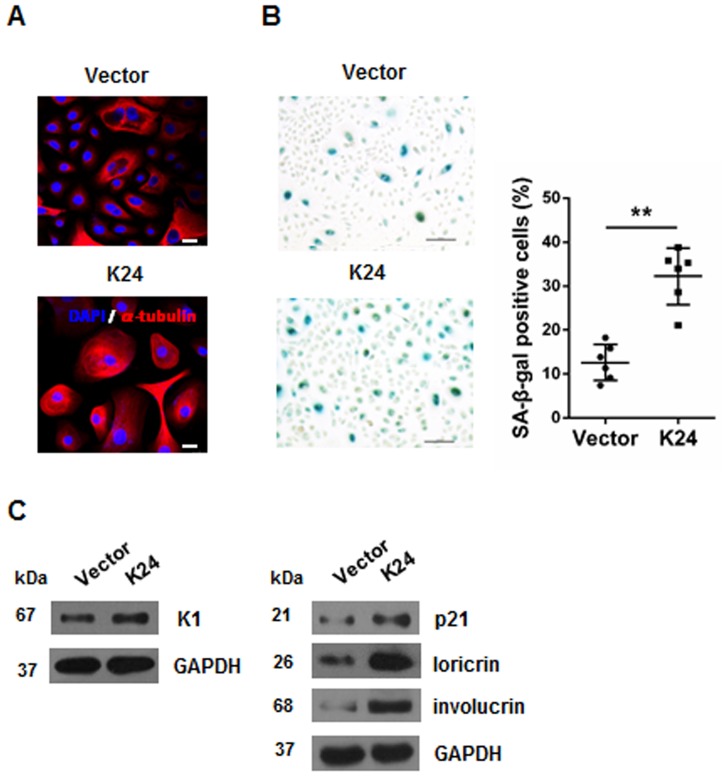
Overexpression of K24 in NHEK induces overexpression of cytoskeletal protein, senescence and differentiation. (A) Confocal analysis showed morphological change of keratinocytes between control and K24 overexpression group by immunofluorescence staining of α-tubulin. Red, α-tubulin; Blue, DAPI; Scale bar = 50μm. Note the increase of stress fibers.The experiment was repeated two times with similar results. (B)Senescence-associated galactosidase activity assay was conducted among control and K24 overexpression group. The values of SA-β-gal positive keratinocytes in two groups are shown in the histogram. n = 6.Similar results were observed in three independent experiments. Student t-test was used,**p<0.01 vs vector control. (C) Effect of K24 overexpression on the levels of K1, loricrin, involucrin and p21 were measured by western blot analysis. The experiment was repeated with primary keratinocytes derived from three different donors.

Since K24 is a cytoskeletal intermediate filament, we assessed whether K24 overexpression could induce aberrant skeleton protein localization or expression. Immunostaining of skeleton protein demonstrated an altered cytoskeleton of α-tubulin with more stress fibers in cytoplasm of K24 transfected cells, which indicating a morphologic change(Fig 3A). SA-β-galactosidase staining showed a 3-folds more positive blue cells in K24 overexpressed keratinocytes than those in control cells (Fig 3B). To evaluate the impact of K24 on keratinocyte differentiation, the expression of epidermal differentiation markers, keratin 1 (K1), loricrin and involucrin were detected by Western blot. The level of K1, loricrin and involucrin was higher in K24 overexpression cells (Fig 3C). Besides, p21, a protein involved in cell cycle inhibition and differentiation, was also increased in K24 overexpressing group compared to the control group (Fig 3C).

### Overexpression of K24 inhibits cell migration of NHEK

**Fig 4:**
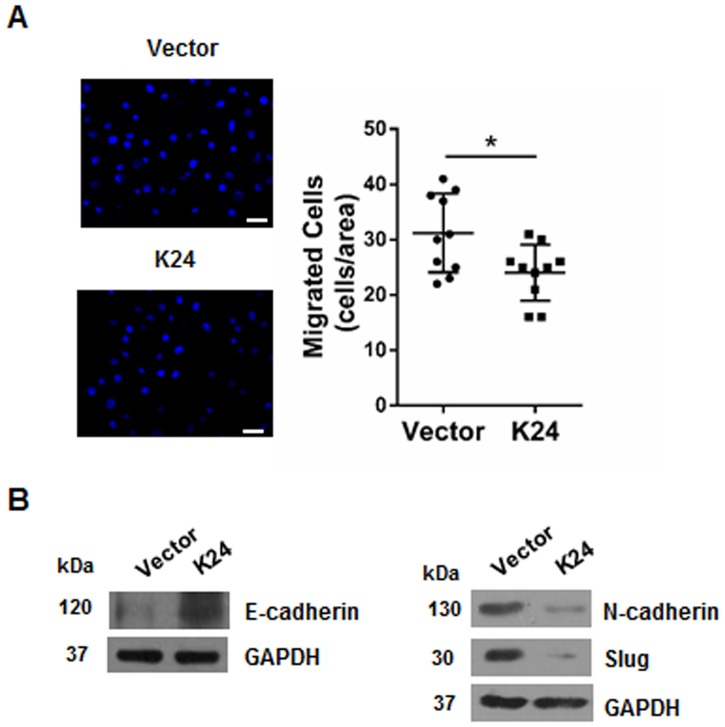
Overexpression of K24 in NHEK inhibits cell migration. (A) Representative images of NHEK cells infected with control or lentiviral vector expressing K24 in transwell migration assays. Right histogram representing the quantification of 10 randomly selected fields after 6 h for the conditions indicated. Similar results were obtained in three independent experiments. Scale bar = 100μm. Student t-test was used, *p < 0.05 vs vector control. (B)Western blot analysis shows the effect of K24 overexpression on the levels of E-cadherin, N-cadherin and slug. GAPDH was used as a loading control. The experiment was repeated with primary keratinocytes derived from three different donors.

The role of K24 in the process of keratinocyte migration was further investigated through transwell assay, which revealed that upregulation of K24 significantly inhibited the migration capacity of NHEK (Fig 4A). Next, the potential effect of K24 on Epithelial-Mesenchymal Transition (EMT) markers was analyzed. K24 overexpression in NHEK resulted in upregulation of the epithelial biomarker E-cadherin. On the contrary, the levels of the mesenchymal biomarkers N-cadherin and slug were decreased by K24 overexpression (Fig 4B).

### K24 overexpression induces autophagy in NHEK

**Fig 5:**
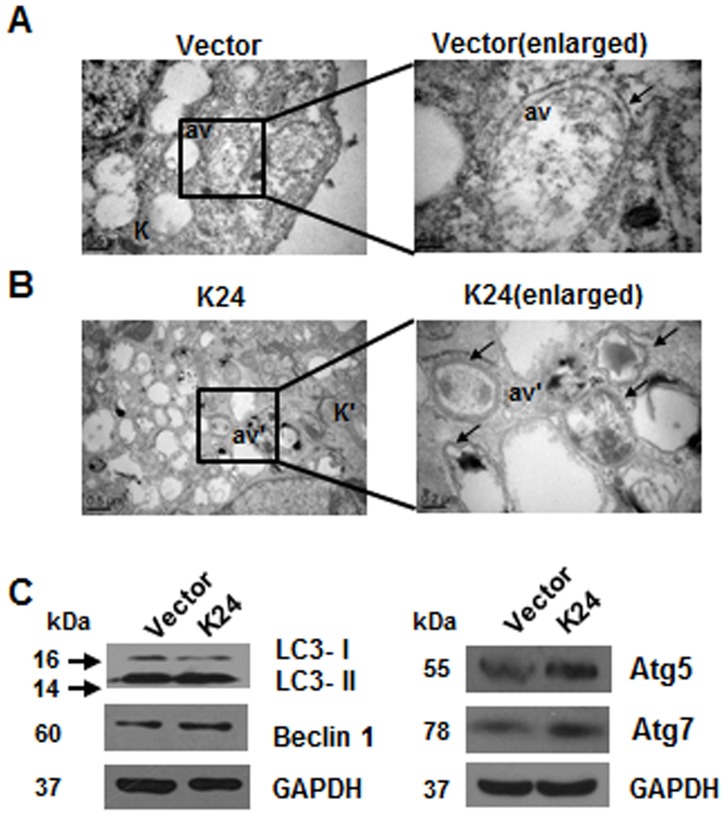
K24 overexpression in NHEK induces autophagy. (A, B) NHEK overexpressing K24 were fixed for TEM analysis comparing with vector control group. The right views (Scale bar = 0.2μm) were the magnification of the region with a black box in the left views (Scale bar = 0.5μm). k: cytokeratin network encircling the cell membrane in good order, av: autophagic vacuoles with a simple or double membrane containing few membranous debris. k': cytokeratin network of cells aggregated disorderly and appearing very rich and contracted. av: a few autophagic vacuoles with residual digested debris. av': numerous autophagic vacuoles with double membrane or isolation membrane containing membranous or non-membranous debris. Blackarrow, double-membrane autophagosome.Similar results were observed in three independent experiments. (C) LC3, Beclin 1 and Atg5 and Atg7 in K24 overexpressing NHEK were detected by western blot using GAPDH as loading control. The experiment was repeated with primary keratinocytes derived from three different donors.

The ultrastructure of K24 overexpressed cells was defined by transmission electron microscopy (TEM). Control keratinocytes displayed an organization with a regular cytokeratin network and several dispersed organelles. There were a few autophagosomes with residual digested debris (Fig 5A). In contrast, K24 overexpressed keratinocytes displayed a highly complicated substructure with multiple autophagosomes and disorganized, compact cytokeratin network. Lots of autophagic vacuoles with double or isolated membrane containing various degraded organelles were observed (Fig 5B). They accumulated in keratinocytes on K24 transfection. Western blotting revealed the conversion of endogenous LC3-I to LC3-II, a marker of autophagosome. Consistent with LC3, Beclin-1, Atg5 and Atg7 were up-regulated in K24 overexpressed keratinocytes (Fig 5C).

## Discussion

As a new subgroup of keratins, the function of K24 in epidermal keratinocytes is yet incompletely understood. The present study demonstrated multiple functions of K24 in proliferation and differentiation. In our investigation, K24 was found to be localized in the upper stratum spinosum and to be more expressed in keratinocytes of larger size. It is widely considered that serial subculture of NHEK resulted in replicative senescence and terminal differentiation[13, 14].Another cellular process may predict the relationship between K24 and differentiation is calcium-mediated signal transduction, a potent inducer of keratinocyte differentiation[15].Here we showed that K24 was up-regulated in senescent keratinocytes upon subculture and CaCl_2_-induced differentiation. These biochemical findings confirmed that both subculture and calcium-inducible differentiation are related to K24 function. It also suggested that K24 overexpressing keratinocytes present a differentiated phenotype.

Accordingly, NHEK with higher K24 protein level revealed some features of differentiated keratinocytes. Their proliferation rate and adhesion ability was reduced. In 2-D culture,K24overexpressing NHEK exhibited low expression of K14, a prototypic marker of dividing basal keratinocytes, a higher content of K1, an early differentiation marker, and increased expression of loricrin and involucrin, markers of exclusively expressed in differentiated suprabasal keratinocytes[7, 16]. Thus, K24 overexpression resulted in a differentiated keratinocyte phenotype and we conclude that it may induced epidermal differentiation. Besides, we demonstrated K24 inhibiting characteristics in view of keratinocytes growth, which may be also supported by its ability to induce cell cycle G1 phase arrest. We found that upregulation of K24 in NHEK cells dramatically arrested the cell cycle during G1 phase by affecting some cell cycle regulators as western blotting showed p21 and p53 were both upregulated in K24 overexpressing keratinocytes. p21 and p53 are important inhibitory components of the cell cycle[17, 18]. Furthermore,p21 also involves in epidermal differentiation[19]. Therefore, increased p21 could contribute to differentiation and senescence of keratinocytes. Although studies suggest that differentiation and apoptosis are two distinct processes, there is also evidence that these processes may overlap and occur concurrently[1, 20]. In our investigation, forced K24 expression induced a significantly higher early apoptosis rate than that of control cells.

Cytoskeleton is one of the most significant cellular mechanical elements, which provides structural stability to the cell undergoing multiple deformations without losing its integrity[21]. Microtubule is the fundamental structural components of cells and is an essential part of the cytoskeleton[22]. It is involved in multiple processes such as cell growth, motility, maintenance of cell shape, cell signal transduction, intracellular transport and mitosis and meiosis[23]. α-tubulin belongs to microtubule and involves in above functions. Our study revealed K24 overexpressing keratinocytes showed more widely cytoplasmic distributed pattern of α-tubulin comparing with the control cells. The control of cell size in mammal is a course of a wide-ranging topic, in which cytoskeletal elements can also play a role in determining size[24]. Gosselin et al. found that senescent keratinocytes displayed the following characteristics: senescence-associated β-galactosidase activity, larger size than young cells, numerous dense particles and several vacuole-like structures of different sizes[25].At the same time, to evaluate the effect of K24 on senescence in NHEK, SA-β-gal activity, a marker for senescence, was determined at 24h after transfection. The result revealed that overexpression of K24increased the number of SA β-gal positive cells threefold when compared with the controls. Accordingly, we found that the key senescence markers, p21, p53 is induced during the senescence mediated by K24.Although the results seem to be contradictory that K24 both involved in differentiation and senescence of primary keratinocytes, there might be some similarities between them. Senescent cells are not quiescent or terminally differentiated cells, although the distinction is not always straightforward[26],however, cellular senescence is characterized by increase in size [27]and cell cycle arrest, leading to irreversible loss of proliferative potential [28].The terminal differentiation of epidermal keratinocytes also involves dynamic morphological changes. During differentiation, keratinocytes undergo a dramatic shape change from small and round to large and flat[29], in addition, cell cycle machinery also play potential roles in terminal differentiation process of epidermal cells which may lead to proliferation inhibition[30, 31].

Our study also showed that overexpression of K24 effectively decreased the incidence of keratinocytes migration. Further, it was showed that K24 overexpression inhibited the metastatic ability of keratinocytes through EMT. EMT is thought to be remodeling of the cytoskeleton, such as down regulation of epithelial keratins, which leads to alterations in cell-to-cell adhesions and changes in polarity and cell motility[32].Loss of keratin expression is a hallmark of psoriasis and skin tumors[33, 34], and EMT is believed to support invasiveness of tumors and metastasis formation[35, 36]. In our study, K24 decreased expression of mesenchymal markers such as N-cadherin and slug, whereas it increased epithelial marker E-cadherin. However, the roles of these transcription factors for the regulation of epithelial/mesenchymal marker expression as well as K24-mediated EMT process remains to be fully investigated.

From what has been discussed, we have shown that K24 induced differentiation, proliferation inhibition, senescence, apoptosis and migration inhibition. Besides, we also demonstrated the ultrastructural structure of K24 overexpressed NHEK by TEM. It was showed that in the presence of K24, the gross morphology of the keratinocytes was affected. There is a compact keratin network arranged in keratinocytes in a disordered way combined with numerous autophagosome formation. It is reported that autophagy can be up-regulated in NHEK and HaCaT cells in vitro by a number of stimuli, such as the induction of differentiation, metabolic stress, and activation of Toll like receptors[37–39].Recently, Yoshihara et al. have reported that autophagy has a significant role in epidermal keratinization and hair growth until a certain stage of maturation[40]. Gosselin et al. reported that senescent keratinocytes may die by autophagic programmed cell death[25].Taken together, our findings have led to the hypothesis that autophagy induced by K24 overexpressing may be involved in keratinocytes differentiation, senescence and apoptosis.

When it comes to the deep mechanism involving in the effects of K24, PKCδ signal pathway was found to be activated in K24 overexpressing keratinocytes. It is reported that PKCδ represents a universal signaling node, and targeting of p53,p38 and caspase3, could overcome multiple mechanisms of resistance against anticancer agents including cell cycle arrest, apoptosis and migration inhibition[41–44].In addition, several studies suggested that PKCδ could be a novel therapeutic target for treatment of several cancer subtypes including breast tumor cells, prostate cancer cells and melanoma tumor cells[45–47].In agreement with these previous reports, our study showed that K24 overexpression inhibited cell proliferation, migration and induced apoptosis, which was accompanied by activation of PKCδ signal pathway. Thus we speculate that functional characteristics altered by K24in NHEK may depend on PKC signaling pathways. However, how this finding relates to the alterations of keratin IF organization, the defects in proliferation and differentiation awaits further study.

Keratinocyte differentiation, which is essential for proper epidermal function, is under control of many signaling pathways leading to changes in expression of many epidermal proteins. Keratin was originally considered to be the primary cell structure support, now it was recognized as multi-faceted effectors in their native context including regulation of cell growth, migration, and apoptosis[48]. We suppose that K24 overexpression induces proliferation inhibition, senescence and apoptosis by autophagic pathways. We conclude that K24 expression pattern may play a vital role in maintenance of epidermis. As a result, this effect can also be viewed as a potent tumor suppressor mechanism. In our study, we did not observe the effect of K24 silencing in NHEK, which indicating the specific effect of K24, however it suggests the possible use of K24 as a novel biomarker of cellular aging, epidermal differentiation and therapeutic application for psoriasis.

## Materials and methods

### Ethics statement

Skin (arm) biopsies (2 mm ×5 mm) were collected from 12 healthy volunteers (6 males and 6 females, 18–55 years old) after obtaining approval by the ethics committee of the Zhejiang University School of Medicine and written informed consent from subjects. In addition to this, all methods applied in experiments were approved by institutional committee and performed in accordance with the relevant guidelines and regulations.

### Cell culture

Primary normal human keratinocytes were established from skin biopsy samples incubated with dispase as earlier described[49]. Briefly, detached keratinocytes free of contamination were seeded onto flasks at a density of 5000 cells/cm^2^ and maintained in Epilife medium supplemented with 0.06 mM calcium, human keratinocyte growth supplement (HKGS, Thermo Fisher Scientific) with media refreshed every 48–72 hrs. Cell differentiation was induced by CaCl_2_. Control cells were incubated with additional 0.03 mM Ca2+, whereas the treated cells were incubated with 1.2 mM Ca2+ for 4 additional days to increase differentiation.

### The virus packaging and generation of the lentiviral particles

All of the vectors including scrambled (pLenti-CMV-2A-GFP-non-target) vectors, the plasmids containing the complete open reading frame (ORF) of human KRT24 gene and 2nd Generation Packaging Mix (pLenti-P2A plus pLenti-P2B) were purchased from Applied Biological Materials Inc., BC, Canada (Abm).The lentiviruses were prepared by transfecting 293T cells with the lentiviral vector and KRT24 Lentiviral Vector plasmids respectively using lentivirus packaging method[50]. The transfection efficiency was evaluated 24 h post-transfection by the percentage of positive green fluorescent protein (GFP) cells observed under a fluorescence microscope. Two days after transfection, the culture supernatant was collected, filtered via 0.45-μm filter and concentrated through ultracentrifugation. The viral particles were aliquoted and used for infection or stored at -80°C for later use.

### The establishment of transduced NHEK

To further understand the roles of K24 in keratinocytes, two transgenic keratinocyte populations were generated and characterized as follows: K24 overexpressing (OE) cells, vector-negative control cells. These genetically modified NHEK provide an in- vitro context for studying K24 function in skin cells. Initially, primary keratinocytes were seeded at 10–20% density in 6-well plate and cultured at 37°C with 5% CO2 overnight. Then, cells were infected with the packaged virus particles and polybrene (6 μg/ml) overnight. The negative control, pLenti-CMV-2A-GPF-non-target vector was simultaneously transduced into the cells. Both of the viruses consistently gave more than 80% of infection efficiency. The infected cells were photographed with the inverted fluorescence microscope (Leica, German).

### Cell viability assay

Cell viability was determined using both MTS assay and EdU proliferation assay. NHEK were seeded (n = 2000 cells per well) in a 96-well microtiter plate and then infected with lentivirus as described above. Twenty-four hours after transfection, the transfected cells were grown in 100 μl complete medium. For MTS assay, briefly, proliferation was studied every 24 h up to a period of 5 d, at which point 100 μl of 0.5 mg/mL MTS solution (Promega) was added to each well and incubated for 3 h at 37°C. Formazan absorbance was read at 490 nm using a plate reader. For EdU proliferation assay, cells were seeded (n = 1× 10^4^ cells per well) in 24-well plates. Transfection of the cells was performed the following day as described above. 24 hours after transfection, the cells were incubated under standard conditions in complete media. Cell proliferation was detected using the incorporation of 5-ethynyl-29-deoxyuridine (EdU) with the EdU Cell Proliferation Assay Kit (Invitrogen). Briefly, the cells were incubated with 50 mM EdU for 3 h before fixation, permeabilization and EdU staining, which were performed according to the manufacturer’s protocol. The cell nuclei were stained with DAPI (Roche) at a concentration of 5ug/ml for 10 min. The proportion of cells that incorporated EdU was determined by fluorescence microscopy (Leica, German).

### Cell cycle and apoptosis assays

NHEK were plated at 20% confluence (1 × 10^5^ cells/well) in six-well plates and infected with lentivirus as described above. After 24 h, cells were incubated under standard conditions in complete media to 90% confluence. Then, cells were harvested and subjected to the following assays. For cell cycle assay, the cells were washed twice with ice cold PBS and fixed in 75% ethanol at -20°C overnight. After fixative removal, cells were incubated with 50 g/mL propidium iodide/RNase staining buffer (BD Biosciences PharMingen, San Diego, USA) at 37°C for 15 min. Flow cytometry analysis of DNA content was performed on a FACScalibur flow cytometer (Becton Dickinson, Franklin Lakes, NJ, USA). For apoptosis assay, cells were stained with AnnexinV-PE/7-AAD kit (BD Biosciences, NJ, USA) and analyzed by flow cytometer. FlowJo software (Version 7.6.1, Treestar, Ashland, OR, USA) was used for subsequent analysis.

### Senescence associated β-galactosidase staining

1×10^4^ NHEK were plated in a 24 well plate and treated as above before the staining procedure. Cells were then fixed with 4% formaldehyde for 15 min at the room temperature. The detection of cellular senescence was performed by using the Senescence Detection Kit (GENMED SCIENTIFICS INC., USA) according to the manufacturer's instructions. Under a light microscopic examination, dark-green colors indicating positive for SA β-Gal activity, suggesting senescence of the observed cells.

### Western blotting

Protein extraction and Western blotting analysis were carried out as previously described[49]. An equal amount of protein (20 μg per well) was loaded and run on SDS—PAGE. The gels were transferred on nitrocellulose filter membrane (millipore) and probed first with the primary antibody. Primary antibodies used were anti-K24(1:500,Abcam, ab180486),anti-K14(1:1000,Santa Cruz, sc-53253), anti-K17(1:1000,Santa Cruz, sc-101931),anti-c-Myc(1:1000,Santa Cruz, sc-70645), anti-p53(1:1000,Santa Cruz, sc-98), anti-Caspase-3(1:500,Cell Signaling Technology, #9662),anti-PKCδ(1:1000, Santa Cruz,sc-213), anti-p-PKCδ(1:1000,Santa Cruz,sc-11770),anti-K1(1:1000,Santa Cruz, SC -17091), anti-loricrin(1:500,Abcam, ab85679), anti-involucrin(1:1000,Santa Cruz, sc-28557), anti-filaggrin(1:1000,Abcam, ab24584), anti-p21(1:1000,Millipore, 04–1039), anti-E-cadherin(1:1000,Santa Cruz, sc-7870), anti-N-cadherin(1:1000,Santa Cruz, sc-7939),anti-slug(1:1000,Cell Signaling Technology, 9585s), anti-LC3A/B(1:500,Cell Signaling Technology, #12741),anti-Beclin-1(1:1000,Cell Signaling Technology, #3495), anti-Atg5(1:1000,Cell Signaling Technology, #12994),anti-Atg7(1:1000,Cell Signaling Technology, #8558),anti-GAPDH(1:2000,Proteintech, 60004-1-lg). Secondary antibodies used in immunoblotting studies were HRP- conjugated (1:5000, Jackson ImmunoResearch). Signals were revealed by enhanced chemiluminescence kit (Millipore).

### Immunofluorescence and laser confocal microscopy

Immunofluorescence was performed according to our previously published work[49].The following antibodies were used for immunoinfluscent assay: anti-K24 (1:200, Abcam, ab87195), α-tubulin (1:200, Santa Cruz, sc-5286). Secondary antibodies labeled with Alexa 488 (green) and cy3 (red) (1:5000, Jackson ImmunoResearch) were used. Cell nuclei were stained with DAPI (Roche) for 10 minutes at a concentration of 5ug/ml (applied after incubation with secondary antibody). Negative controls stained with secondary antibody alone or Rabbit IgG showed no immunolabelling. The K24 location in keratinocytes and epidermis were detected using an inverted fluorescence microscope (Leica, German). The α-tubulin cytoskeletons of lentivirus-infected keratinocytes were visualized using a confocal laser scanning microscopy (Leica, SP8, Germany).

### Transwell migration assay

We assessed whether overexpression of K24 induces chemotaxis of the keratinocytes using a transwell migration assay. Specifically, NHEK were plated at 20% confluence (1 × 105 cells/well) in six-well plates and infected with lentivirus as described above. After 24 h, cells were incubated under standard conditions in complete media. After reaching 90% confluence, cells were harvested and utilized for transwell migration assays. A 24-well plate containing an 8-mm-pore size chamber insert (BD Biosciences, Franklin Lakes, NJ, USA) was used to evaluate the migration of keratinocytes. The upper transwell chamber was seeded with control or K24 transfected keratinocytes (∼2.5×10^5^) resuspended in 100 μl of Epilife medium containing 0.1% bovine serum albumin. In each lower chamber, 500 μl Epilife medium containing keratinocyte growth factor was added as a chemoattractant. After 6h of incubation at 37°C, the upper part of the membranes was swabbed to remove non-migrated cells, the cells that had migrated through the pore were fixed by formalin and stained with 5μg/ml of DAPI (Roche) for 10 min, and its fluorescence signal was observed under an inverted fluorescence microscope.

### Transmission electron microscopy (TEM)

To deeply investigate the ultrastructural alterations of NHEK by K24 overexpressing, we further investigated the fine structure of transfected NHEK by TEM. K24 overexpressed keratinocytes was analyzed by TEM by comparison with control cells. The lentivirus-infected cells were harvested and washed with PBS and then fixed in ice-cold 2.5% glutaraldehyde overnight. Cell pellets were dissected, cut into 1–2 mm2 pieces. After being washed with PBS three times for 15 min, cells were postfixed in 1% OsO4 for 1 h and stained with 2% uranyl acetate for 30 min at room temperature. Then cells were dehydrated through a graded series of ethanol (50, 70, and 90%) for 15 min each, ethanol (100%) for 20 min, and 100% acetone for 20 min, respectively. Sections of 70-nm thickness were placed on copper grids (Leica) and imaged using a JEM 1200EX transmission electron microscope (JEOL, Tokyo, Japan).

### Statistical analysis

GraphPad Prism 5 software (Graphpad Software, Inc., La Jolla, CA, USA) was used to perform statistical analysis. Student’s *t*-test was used. All data are presented as mean ± SEM. Asterisks denote statistical significance (* P<0.05; ** P<0.01).

## Supporting information

S1 Fig Negative controls for immunofluorescent staining of normal keratinocytes and epidermis. No significant staining was detected when the primary antibody was used Rabbit IgG instead of K24 antibody for the immunofluorescent staining procedure.
(TIF)

S2 Fig Enlarged image of Fig 1B. Immunofluorescent staining of normal epidermis by K24 antibody. SC,stratum corneum.G,granular layer. Scale bar = 50 μm.
(TIF)
