# Supplementary figures and images for: Role of keratin 24 in human epidermal keratinocytes
Source: PLoS One. 2017 Mar 31;12(3):e0174626. doi: 10.1371/journal.pone.0174626 (PMC5376294; doi:10.1371/journal.pone.0174626)

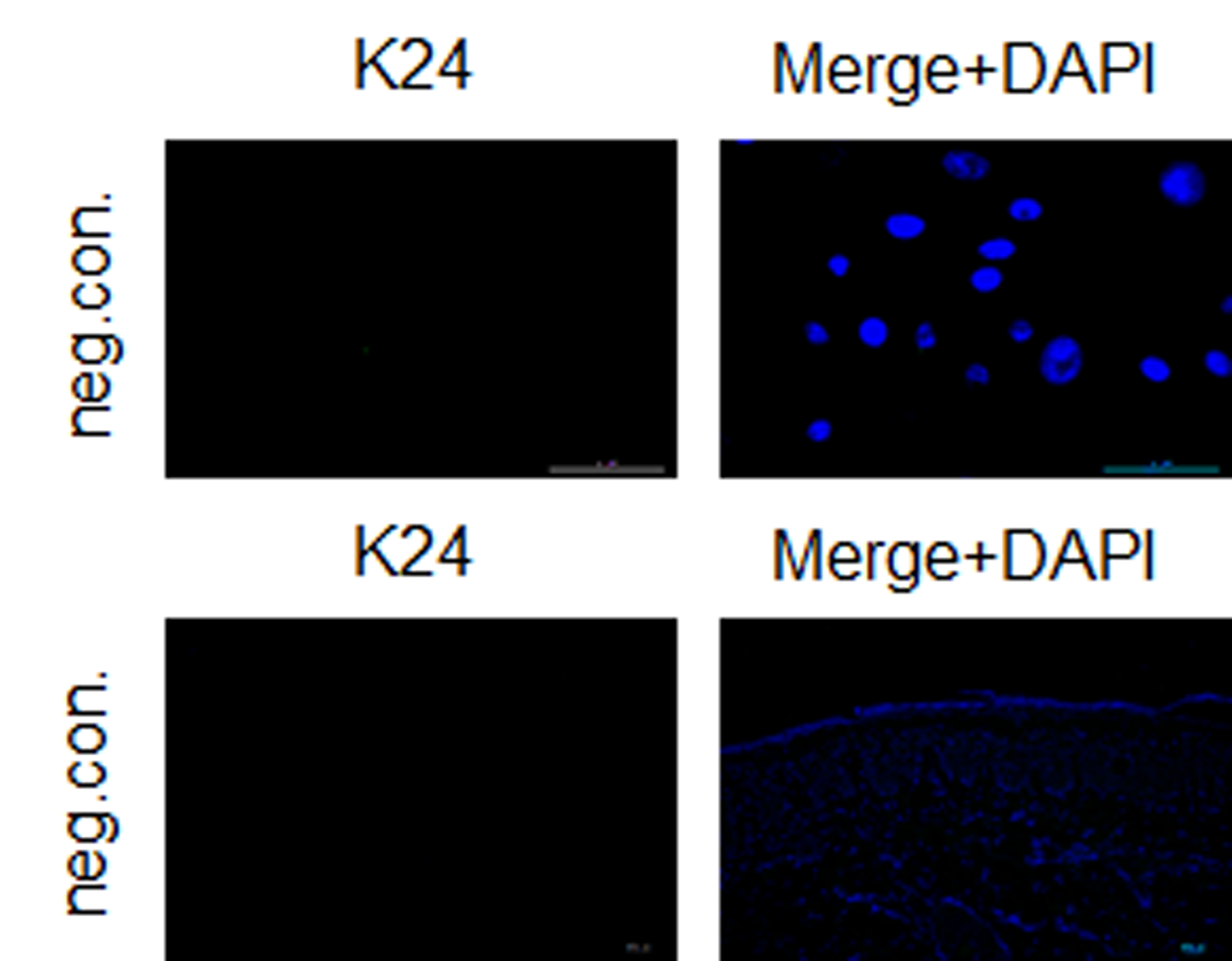

Supplement: S1 Fig — No significant staining was detected when the primary antibody was used Rabbit IgG instead of K24 antibody for the immunofluorescent staining procedure. (TIF) [file pone.0174626.s001.tif]

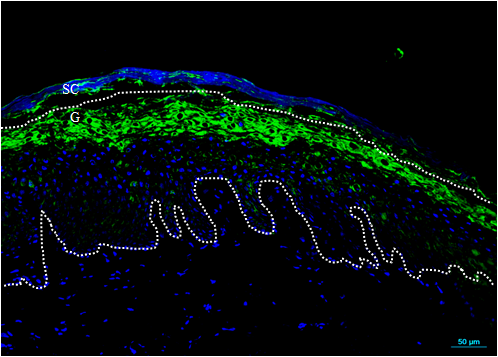

Supplement: S2 Fig — Immunofluorescent staining of normal epidermis by K24 antibody. SC,stratum corneum.G,granular layer. Scale bar = 50 μm. (TIF) [file pone.0174626.s002.tif]
